# Bioinformatics analysis of glial inflammatory responses to air pollution

**DOI:** 10.1186/s12974-017-0937-z

**Published:** 2017-08-15

**Authors:** Chenyu Li, Wei Jiang, Nina Tang, Yan Xu

**Affiliations:** Department of Nephrology, The Affiliated Hospital of Qingdao University, 16 Jiangsu Road, Qingdao, 266003 China

Dear editor:

We read with great interest the article by Dr. Woodward and colleagues [1], “Toll-like receptor 4 in glial inflammatory responses to air pollution in vitro and in vivo” which appeared in the 15 April 2017 of Journal of Neuroinflammation. Since the results of the article is very attractive for us, we collected original data from NCBI (https://www.ncbi.nlm.nih.gov/pmc/articles/PMC5391610/bin/12974_2017_858_MOESM1_ESM.zip) which has been submitted by Woodward et al. and used different methods to perform the bioinformatics analysis in each group; however, we get different results against the article, and we think the author’s methods in bioinformatics analysis are inappropriate (Fig. 1).

**Fig. 1:**
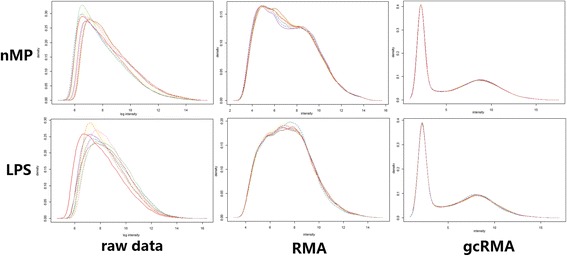
The difference of normalization between RMA and gcRMA

We noticed that the author used significance analysis microarrays (SAM) in differentially expressed gene (DEG) analysis which, usually, cause high false positives. We utilize a fewer false positives method, Limma (Linear Models for Microarray Analysis) [2] package, which was widely used statistical tests to obtain differential expression based on R programming language and more accurate than SAM. The results showed 572 RNAs (contain 22 LncRNAs) were differentially expressed between the “control cultures” and “nPM-treated” groups (FDR < 0.05, |logFC| > 1), and 1931 RNAs (contain 147 LncRNAs) were differentially expressed between the “control cultures” and “LPS-treated” groups (FDR < 0.05, |logFC| > 1) (Additional files 1 and 2). Due to the high false positives, the number of DEGs which were detected by SAM are more than our results. So for high-level analysis, we suggest using Limma or combining more than one method and then only taking the common genes of all methods to get more accurate results [3].

 Moreover, for preprocessing microarray data, we recommend using GeneChip Robust Multi-array Averaging (gcRMA). We found that the author used RMA method which results of normalization are very close to gcRMA [4], but gcRMA is an improved algorithm of RMA that can be used to achieve a more accurate expression of the gene chip probes by using sequence-specific probes (Fig. 1). Attached figure shows that the gcRMA is better than RMA in raw data normalization.

## Additional files

Additional file 1: Differentially expressed RNAs between “LPS-treated”and control groups. (CSV 50 kb)

Additional file 2: Differentially expressed RNAs between “nPM-treated”and control groups. (CSV 167 kb)

## Abbreviations

DEG: Differentially expressed genes
FDR: False discovery rate
gcRMA: GeneChip Robust Multi-array Averaging
Limma: Linear models for microarray analysis
LncRNA: Long non-coding RNA
LPS: Lipopolysaccharide
NCBI: National Center for Biotechnology Information
nPM: Nanoscale particulate matter
RMA: Robust Multi-array Averaging
SAM: Significance analysis microarrays

## References

[1] Woodward NC, Levine MC, Haghani A (2017). Toll-like receptor 4 in glial inflammatory responses to air pollution in vitro and in vivo. J Neuroinflammation.

[2] Ritchie ME, Phipson B, Wu D (2015). Limma powers differential expression analyses for RNA-sequencing and microarray studies. Nucleic Acids Res.

[3] Chrominski K, Tkacz M (2015). Comparison of high-level microarray analysis methods in the context of result consistency. PLoS One.

[4] Giorgi FM, Bolger AM, Lohse M (2010). Algorithm-driven artifacts in median polish summarization of microarray data. BMC Bioinf.

