# Supplementary material for: Bioinformatics analysis of glial inflammatory responses to air pollution
Source: J Neuroinflammation. 2017 Aug 15;14:160. doi: 10.1186/s12974-017-0937-z (PMC5558762; doi:10.1186/s12974-017-0937-z)
Supplement: Related texts

# Reply: Authors’ response

**Authors:** Morgan Levine, Nicholas Woodward, Caleb Finch

Dear Dr. Li,

While we recognize the issues surrounding false discoveries, we do not agree with your comment suggesting “that the methods in bioinformatics analysis [employed in our study] are inappropriate”. We are not surprised that your Limma bioinformatics findings differed slightly from SAM in relation to the number of responding genes, which of course varies by method and criteria, because of this reasoning that we tested the robustness of our results using a variety of distinct approaches, rather than relying on any one in particular. Further, our conclusions pointing to the importance of the TLR4 pathway are not based on the number of differentially expressed genes—which you imply may be biased—but rather are substantiated by findings derived from state-of-the-art systems biology approaches. For instance, the set of differentially expressed genes based on SAM (which we acknowledge may include false positives) was further analyzed using pathway enrichment and gene ontology analysis. Similarly, our findings were further substantiated using WGCNA—a network analysis approach that identifies higher order modules, rather than individual genes, and thus has been shown to be more robust when it comes to gaining biological insight from high-dimensional data. Finally, and perhaps most importantly, we successfully validated our findings with further experiments both in vitro and in vivo.
